# Surface-Bound Humic Acid Increased Propranolol Sorption on Fe_3_O_4_/Attapulgite Magnetic Nanoparticles

**DOI:** 10.3390/nano10020205

**Published:** 2020-01-24

**Authors:** Yuehua Deng, Yani Li

**Affiliations:** 1 College of Geology and Environment, Xi’an University of Science and Technology, Xi’an 710054, China; 2 Shaanxi Provincial Key Laboratory of Geological Support for Coal Green Exploitation, Xi’an 710054, China

**Keywords:** humic acid, Fe_3_O_4_/attapulgite, propranolol, sorption

## Abstract

This study explored the feasibility of utilizing a novel sorbent humic acid (HA) coated Fe_3_O_4_/attapulgite (MATP) magnetic nanoparticles (HMATP) for the sorption of propranolol from aqueous solutions. MATP and bare Fe_3_O_4_ nanoparticles were also synthesized under similar preparation conditions. The FTIR, Zeta potential, XRD, VSM, TEM, and TGA analyses were conducted to characterize the sorbent materials. The effects of pH, sorbent dosage, ionic strength, HA in the aqueous solution, contact time and initial sorbate concentration on sorption of propranolol were investigated using batch sorption experiments. The results suggested that the sorption capacity of HMATP showed little change from pH 4 to 10. Na^+^ and Ca^2+^ slightly inhibited the sorption of propranolol on HMATP. While HA in solution enhanced both MATP and HMATP, which indicated that HMATP can resist HA interference in water. Further, the less leaching amounts of Fe and HA suggested a good stability of HMATP. In all conditions, sorption capacity of propranolol on HMATP was obviously higher than that on MATP, which indicated that surface-coated HA played an important role in the propranolol sorption process. Electrostatic interaction, cation exchange, hydrogen bonding, and π–π electron donor acceptor interactions were considered as the sorption mechanisms.

## 1. Introduction

Currently, cardiovascular disease is one of the leading causes of death in China. Findings from the Chinese National Center for Cardiovascular Diseases show that one in five Chinese are in poor cardiovascular shape, and that one person dies from heart disease every ten seconds in China. Consequently, various cardiovascular drugs have been extensively used in the past few decades. Beta-blockers, or β-blockers, are predominantly used to treat cardiovascular disease [1,2]. Their widespread application, as well as incomplete removal during wastewater treatment [3], result in them occurring in various aquatic environments [2,4,5,6,7]. Propranolol has been detected extensively in the water compartment at low concentrations in the ng/L range as it is a commonly used β-blocker [2,8,9]. Although designed for human use, more and more evidence has indicated that propranolol may pose harm to aquatic organisms [10,11,12,13,14,15], it must be pointed out that propranolol has shown high hydrolytic stability [16]. Meanwhile, propranolol may bioaccumulate in water ecosystems due to its lipophilicity [2,3,17]. Therefore, it is urgent that various efficient methods are developed to remove propranolol from water.

Sorption is generally considered to be a simple, relatively low-cost and effective method in removing the low concentration of pollutants from water. Over the past decade, sorption has been applied to remove beta-blockers from water with various sorbents [18,19,20,21,22,23,24,25,26,27]. Among these sorbents, attapulgite has been considered as a promising purification material to contaminated water, as it has a high surface area, strong sorption capability, and is abundant in nature [27,28]. In our previous work, we studied the ability of natural attapulgite, acid treatment attapulgite (marked as ATP), KH550 modified attapulgite, and chitosan modified attapulgite to remove propranolol from water. The results showed that propranolol was more effectively removed by the acid treatment attapulgite compared with the other three sorbents, mainly due to the fact that ATP carried more negative charges [27]. However, the attapulgite cannot easily be recycled from water after sorption due to the difficulty in solid/liquid separation in practical application. Magnetic separation technology has been used to solve this problem. Liu et al. prepared attapulgite/Fe_3_O_4_ magnetic nanomaterials by co-precipitation technique and the results showed that the more Fe_3_O_4_ nanoparticles loaded the better magnetic separation performance possessed [29]. Meanwhile, magnetic attapulgite was also synthesized in our previous study to remove propranolol with an excellent magnetism [30]. A solution of the separation problem brings new problems. On the one hand, Fe_3_O_4_ nanoparticles are easily oxidized and aggregated. On the other hand, the sorption capacity of magnetic attapulgite decreases because of the neutralization between the positive charge of Fe_3_O_4_ nanoparticles and the negative charge of attapulgite. Coating of natural organic matter (NOM) on materials has been proven to potentially enhance the sorption capacity and stabilize the magnetic nanoparticles [31].

As a ubiquitous NOM in a natural aquatic environment, humic acid (HA) possesses a skeleton of alkyl and aromatic units that attach with carboxylic acid, phenolic hydroxyl, and quinone functional groups [32,33]. The existence of oxygen-containing functional groups can promote HA complexion with various organic contamination. Many studies have reported the application of HA-modified materials as sorbents. Iglesias et al. suggested that goethite coated with HA showed higher paraquat sorption capacity than bare goethite [34]. Similarly, Guo et al. investigated that HA-goethite complexes promoted the photolysis and sorption of tylosin and sulfamethazine, which may be ascribed to the heterogeneous surface of HA [35]. Radwan et al. prepared humic acid-carbon hybrid materials to remove phenol, 2,4,6-trichlorophenol (2,4,6-TCP) and atrazine, and the results showed that the modified composite had higher sorption ability than the pure materials [36]. Rashid et al. treated phosphate in aqueous solution by modified magnetic nanoparticles with a natural protective coating of HA which interacted with the magnetic nanoparticles by surface complexation ligand exchange reactions to increase the sorption ability of sorbent [31]. Similar materials were prepared by Singhal et al. to extract uranium from sea water, and the sorption capacity increased with the increase in HA [37]. In our previous work, the effect of HA on propranolol sorption on ATP was conducted and the results showed that HA, in solution, had a solubilizing effect on propranolol [27], which suggested that HA itself had a strong affinity to propranolol. Taking the above results into consideration, it is reasonable to assume that combining HA with the surface of magnetic attapulgite will promote propranolol sorption and prevent oxidation and aggregation of magnetic nanoparticles.

In this study, magnetic attapulgite (MATP) and surface-coated MATP with different masses of HA were developed for the removal of propranolol from water. The main aims of our work were to determine the changes in physicochemical properties of prepared material caused by the interactions of MATP and HA, to quantify the increase of propranolol sorption on MATP as the result of the surface-bound HA, and to explore the underlying sorption mechanisms. Various methods were used to characterize the above materials. Different factors including pH, dosage, ionic strength and HA in solution were also investigated in detail. The results of this study are expected to offer new insight into how MATP interacts with HA for the removal of propranolol from real water environments.

## 2. Materials and Methods 

### 2.1. Materials

**Figure 1:**
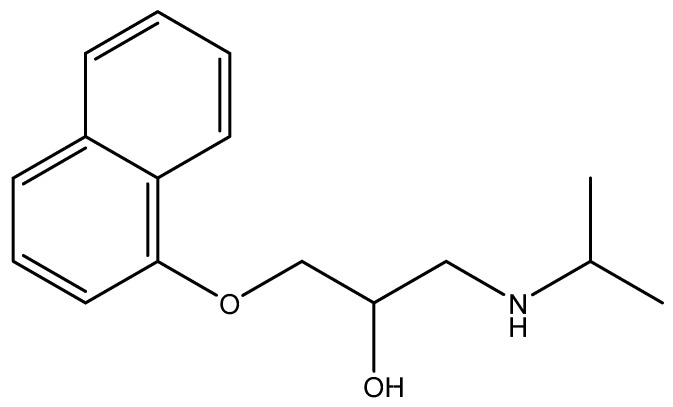
The structural formula of propranolol.

Attapulgite used was taken from Xuyi (China) and purified by 5% HCl solution to remove carbonate and sorbed cations, as described in our previous work [27]. Acid-treatment attapulgite was marked as ATP. Propranolol hydrochloride (Figure 1) used was obtained from Acros (USA). Commercial sodium salt of humic acid, from Sigma-Aldrich Chemie GmbH (Germany), was used without further purification. The high performance liquid chromatography (HPLC) grade of methanol and acetonitrile were used for determination of propranolol content. Twice-distilled water was employed in batch experiments.

### 2.2. Synthesis of HA Coated MATP Magnetic Nanoparticles

HA coated MATP magnetic nanoparticles were synthesized based on methods modified from reference [38]. Three different dosages (0.5 g, 1.0 g, and 1.5 g) of HA coated with MATP were used. HMATP was used to represent MATP with HA load of 1.5g. Firstly, 1.0 g of ATP, 4.0 g of FeSO_4_·7H_2_O and 200 mL of deionized water were added into a 250 mL conical flask with a stopper. Afterwards, the suspension was then heated until the temperature reached 90 °C. Secondly, 0.5 g, 1.0 g, and 1.5g of HA were separately added into 80 mL aqueous solution containing 3.6 g of NaOH and 1.8 g of NaNO_3_, and the three solutions were further added dropwise into the ATP-metal suspension under nitrogen protection. After that, the temperature was kept at 90 °C for 4 h. The precipitates were isolated by a permanent magnet and washed to neutral. In addition, HA composites were washed until the presence of HA in washing solution was not detected on the UV-vis spectrometer (Shimazu, Japan). For comparative purposes, MATP was also prepared in the same way but without HA. Moreover, Fe_3_O_4_ magnetic nanoparticles were synthesized using a similar method. The obtained precipitates were dried in a vacuum oven for further use. 

### 2.3. Characterization of the As-Made Magnetic Nanoparticles

The changes of functional groups on materials were analyzed by FTIR (Nicolet 5700 FTIR spectrometer, Thermo Nicolet, USA). The morphology of HMATP was characterized by TEM (JEM-2100 TEM, JEOL, Japan). The crystal structures of the sorbents were analyzed by their XRD patterns, measured utilizing an X-ray diffractometer (ARL Co., Switzerland). A vibrating sample magnetometer (VSM, LAKESHORE-7304, USA) was used for characterizing the magnetic properties of Fe_3_O_4_, MATP, and HMATP at room temperature. Thermo gravimetric (TG) analysis was carried out to determine the thermal stability and degree of organic coating, conducted with a thermogravimetric analysis (Perkin-Elmer Pyris 1, USA). The surface potential charges of the sorbents were measured by using a zeta potential analyzer at different pH values (Brookhaven Instruments Corp., USA). The stability of HMATP was analyzed by the leaching content of iron and HA which was measured by atomic absorption spectrophotometer (Thermo Fisher Scientific, USA) and UV-Vis spectrometer (Shimazu, Japan) at 254 nm, respectively.

### 2.4. Sorption Experiments

All propranolol sorption experiments were obtained using a batch equilibration technique at room temperature. The pH of the reaction system was adjusted with a small amount of HCl and/or NaOH solution and kept at pH 5.8–6.0, except for the pH effect test. Next, 0.04 g of HMATP and MATP were added to tubes which contained different concentrations of propranolol solution in the range of 5 to 250 mg/L to evaluate propranolol sorption isotherm. Except for sorption isotherm, the initial concentration of propranolol was maintained at a constant value of 25 mg/L. The impact of the MATP and HMATP doses on propranolol sorption was explored in the range of 0.01–0.12 g. NaCl and CaCl_2_ solutions were employed in ionic strength tests (both in the range of 50–500 mg/L) with 0.04 g sorbents. Different contact time (5 min–21 h) was controlled for the sorption kinetic test. The pH value of solution was changed from 4 to 11 to investigate the difference in the propranolol sorption. The effect of HA in aqueous solution on propranolol sorption was studied in the range from 5 to 40 mg/L. 

After centrifugation, the supernatant was collected to determine propranolol concentration by HPLC (Agilent 1200) with a diode array detector and a C18 reversed phase column (4.6 × 250 mm^2^, 5 μm, Agilent). The mobile phase consisted of 30:70 acetonitrile: 0.1% formic acid with a flow rate of 1 mL/min at 290 nm wavelengths. The equilibrium sorption amounts of propranolol were calculated according to Equation (1):

(1) $$q_{e}=\frac{(C_{0}-C_{e})V}{m}$$

where *q_e_* is the amount of propranolol sorbed at equilibrium. *C_0_* represents the initial propranolol concentration and *C_e_* is the propranolol concentration after sorption. *V* is the volume of propranolol solution, and *m* is the materials dose. 

## 3. Results and Discussion

### 3.1. Material Characterization

**Figure 2:**
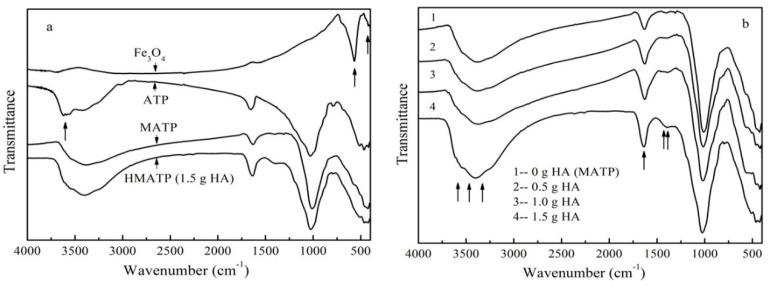
(**a**) FTIR spectra of Fe_3_O_4_, ATP, MATP and HMATP. (**b**) FTIR spectra of sorbents with different coating amount of HA.

The FTIR spectra of Fe_3_O_4_, ATP, MATP, and HA coated Fe_3_O_4_/ATP magnetic nanoparticles with different coating amount of HA are shown in Figure 2. From Figure 2a it can be seen that both MATP and HMATP maintain a skeleton structure of ATP. The characteristic peak at 3600 cm^−1^ in ATP is associated with the stretching mode of the OH^−^ group. The same peak in Fe_3_O_4_ was not obvious. It is speculated that the above peak in MATP disappears because of an overlap of Fe_3_O_4_ and ATP. Fe^2+^–O^2−^ can be observed at the characteristic peaks at 567 cm^−1^ of Fe_3_O_4_, which is consistent with reference [29]. The same peak is show in HMATP and MATP, rather than ATP, which means that the surface of the two materials has been occupied with Fe_3_O_4_ nanoparticles. FTIR spectra (Figure 2b) show the C=O stretches of the three HA coated Fe_3_O_4_/ATP magnetic nanoparticles are at about 1635 cm^−1^. Moreover, the band becomes stronger with the increase of HA dosage, suggesting the carboxylate anion reacts with the FeO surface, due to the fact that the C=O stretches in free carboxylic acid are above 1700 cm^−1^ [33]. As also seen in Figure 2b, the band of the three HA coated Fe_3_O_4_/ATP magnetic nanoparticles at 1393 cm^−1^ become stronger, compared with the spectrum of MATP, this is mainly due to the CH_2_ scissoring of HA. Last but not least, the differences in the spectra of MATP and the three HA coated Fe_3_O_4_/ATP magnetic nanoparticles are the bands at about 3500 cm^−1^ which become stronger and broader, probably due to the strong and broad sorption band of the hydrogen-bonded hydroxyl groups of HA. The changes of FTIR peaks show the successful attachment of HA and MATP. 

**Figure 3:**
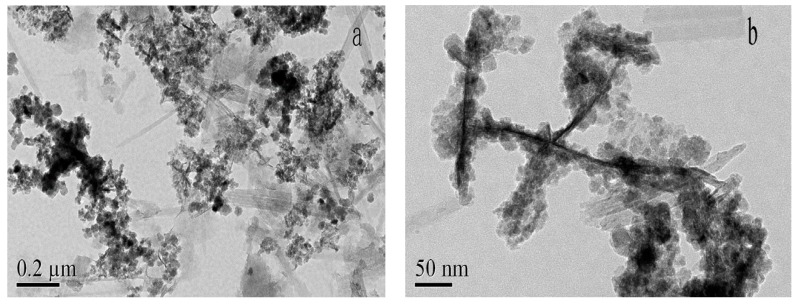
TEM images of HMATP (**a**) at 0.2μm and (**b**) at 50nm.

The TEM images of HMATP are exhibited in Figure 3. Attapulgite has a length of about 400 nm and a diameter of 20 nm and exists in a single rod shape (Figure 3a). Many rods are interwoven into a network or polymerized, due to van der Waals forces. Black spheres, grown along a single rod shape of ATP, represent Fe_3_O_4_ and HA in Figure 3b. Fe_3_O_4_ nanoparticles, with a diameter of about 20 nm, showed the regular cubic crystal structure in our previous study [38]. The black balls presented, rather than cubic structures indicate that Fe_3_O_4_ nanoparticles are surrounded by HA, due to electrostatic attraction. A small amount of HA also adheres to the ATP surface, which is in accordance with the report [32].

**Figure 4:**
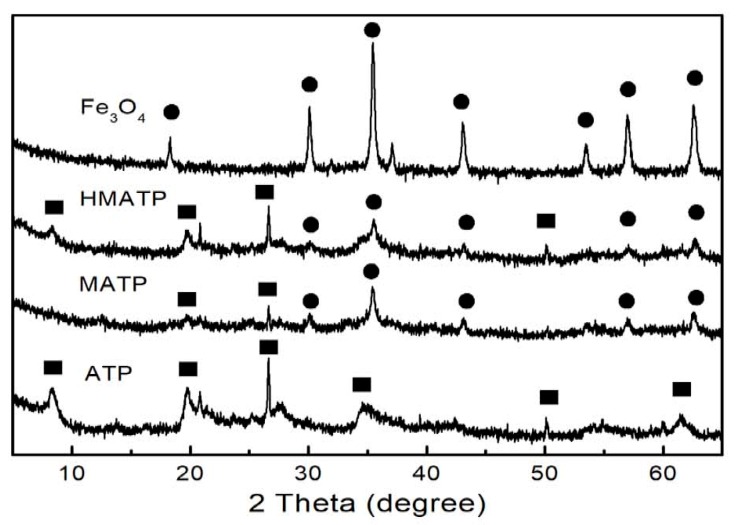
XRD patterns of Fe_3_O_4_, ATP, MATP and HMATP.

The XRD patterns of Fe_3_O_4_, ATP, MATP and HMATP are depicted in Figure 4. It can be observed that some diffraction peaks are obtained at 2θ = 8.3°, 13.6°, 19.7° and 27.28° in the ATP curve. Xue et al. showed that the above peaks represent the (110), (200), (040) and (400) planes of the attapulgite [39]. At the same time, the typical peaks of pure Fe_3_O_4_ are also observed and consistent with the reference [40]. In curves of MATP and HMATP, the characteristic diffraction peaks of magnetite Fe_3_O_4_ and ATP are present in both MATP and HMATP, indicating that Fe_3_O_4_ nanoparticles have been coated onto the surface of ATP. It should be pointed out that HA cannot be distinguished by XRD, because it does not diffract.

**Figure 5:**
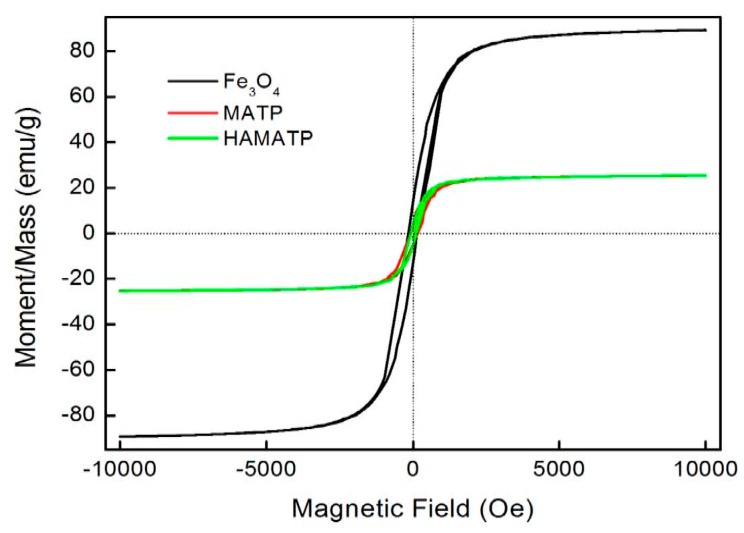
Magnetization curves of Fe_3_O_4_, MATP and HMATP.

The magnetization curves for the Fe_3_O_4_, MATP and HMATP nanocomposites are shown in Figure 5. The curves exhibit that the saturation magnetization of pure Fe_3_O_4_ nanoparticles is 89.03 emu/g. However, due to the existence of ATP, the saturation magnetization of MATP decreases to 25.24 emu/g. In addition, compared with MATP, HMATP has a comparable saturation magnetization (25.36 emu/g), which means that the attachment of HA does not affect the magnetism of MATP on the whole. Further, the hysteresis loops of the two sorbents are very narrow and the magnetic coercive force is small, indicating that the samples are soft magnetic materials. This result indicates that both MATP and HMATP can be easily separated from water after use, by the external magnetic field.

**Figure 6:**
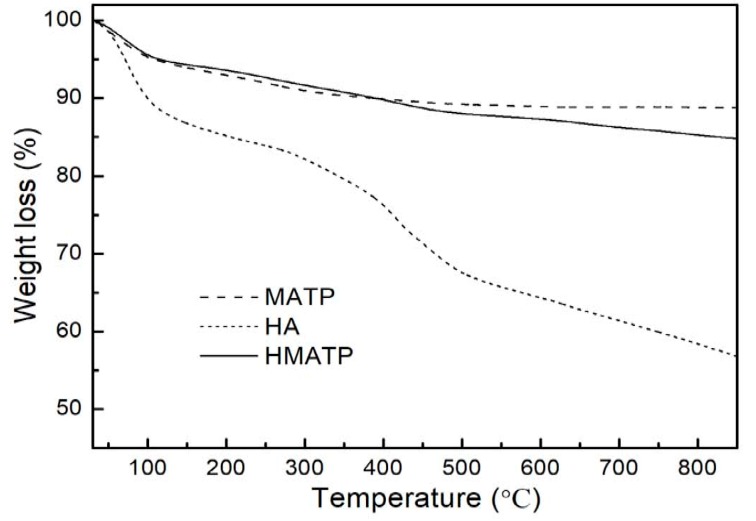
Thermogravimetric curves of HA, MATP and HMATP.

Figure 6 displays the thermogravimetric profiles of HA, MATP and HMATP. As seen from Figure 6, HA is not thermally stable when the temperature was increased, and about 32% of the sample decomposes before 500 °C. In addition, MATP has a higher thermal stability than HA. After HA coating, percentage weight loss is more in HMATP as compared to MATP, and about 8% of HA has been loaded onto MATP.

### 3.2. Effect of pH

**Figure 7:**
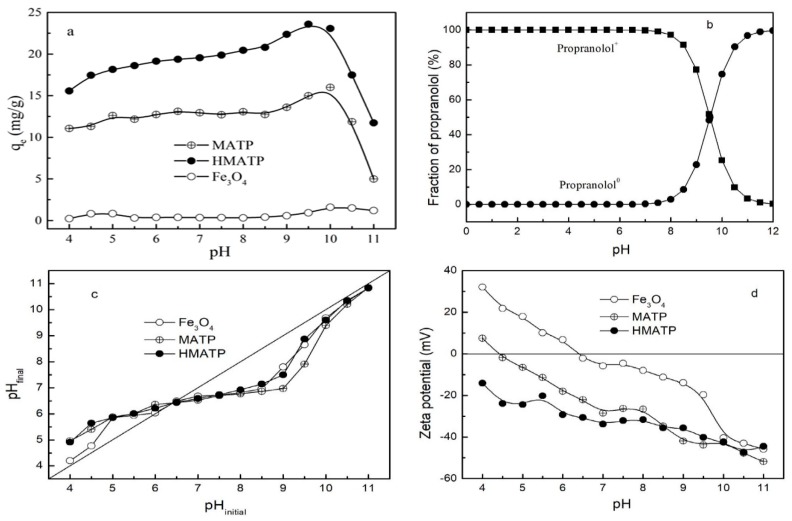
(**a**) Effect of initial pH on the sorption of propranolol on Fe_3_O_4_, MATP and HMATP. (**b**) The pH-dependent speciation of propranolol molecule. (**c**) The variation tendency of solution pH before and after sorption. (**d**) The zeta potential of Fe_3_O_4_, MATP and HMATP.

The property of the sorbents and the species of propranolol are affected by solution pH. With this in mind, the influence of the initial pH on the performance of Fe_3_O_4_, MATP and HMATP was assessed in the pH range 4–11. As Figure 7a shows, the sorption capacities of propranolol on MATP and HMATP increase slowly at the beginning of the sorption, and then decrease sharply as pH increases further, but Fe_3_O_4_ has almost no sorption to propranolol at tested pH range. Propranolol contains a secondary amine group which can be hydrolyzed when the environmental pH is lower than pKa (9.53) [41,42], therefore the species of propranolol present in water are mainly positively charged when initial solution pH ranges from 4 to 9.53 (Figure 7b). The same type of sorbates will occupy the same type of sorption sites. When pH < 6, MATP and HMATP have shown lower relative sorption capacity to propranolol, which may be ascribed to competition between H^+^ and propranolol. The phenomenon is also reflected by the pH value changes. The pH values after sorption equilibrium were higher than those before sorption, which suggests cation exchange is an important sorption mechanism (Figure 7c). MATP is positively charged when pH < 4.5 (Figure 7d), electrostatic repulsion between cationic propranolol and MATP is responsible for the low sorption capacity. For pH values increasing from 6 to 8.5, or after coated with HA, an enhanced electrostatic attraction between drug and sorbents is caused by a more negative zeta potential. The sorption capacity of MATP and HMATP rapidly increases at pH from 8 to 10, reaches maximum around pH = pKa, and sharply decreases when pH is greater than 10. As shown in Figure 7b, the fraction of cationic propranolol decreases from 97.13% to 0.34% and the propranolol molecule sharply increases from 2.87% to 99.66% at the pH range from 8 to 12. When pH > 10, electrostatic interactions have little effect on the sorption process due to the neutral molecule of propranolol. On the other hand, Figure 7a also displays bare Fe_3_O_4_ and shows slight sorption capacity for propranolol in the pH range of 4 to 11 on the whole. However, the sorption affinity of MATP to propranolol is much stronger than bare Fe_3_O_4_ nanoparticles, indicating that the high sorption capacity of propranolol on MATP is attributed to the role of ATP. Similarly, HMATP has better sorption ability for propranolol than MATP at the working pH range, but the zeta potential of MATP is near to that of HMATP, as shown in Figure 7d. This observation suggests that HA, in the surface of HMATP, may also play a key part in propranolol sorption.

### 3.3. Effect of Ionic Strength

**Figure 8:**
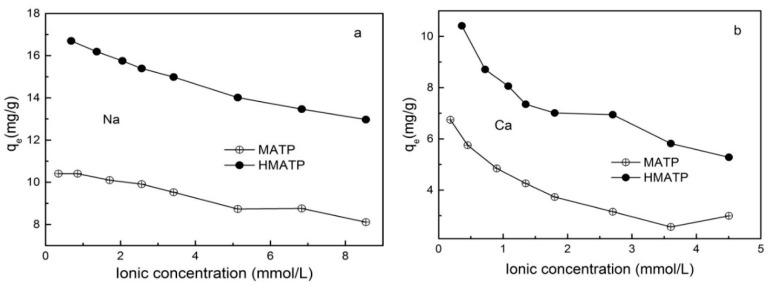
Effect of (**a**) Na^+^ and (**b**) Ca^2+^ on the sorption of propranolol on MATP and HMATP.

A large number of inorganic cations in water will affect the migration of pollutants. Figure 8 exhibits the effect of ionic strength on propranolol sorption on MATP and HMATP. As seen in Figure 8, propranolol sorption, on both MATP and HMATP, decreases with the increase of Na^+^ and Ca^2+^, and the divalent Ca^2+^ ions have more significant effects on the sorption capacity of propranolol than monovalent Na^+^ ions. The sorption competition between the cations and cationic propranolol for the same sorption sites explains the decrease of propranolol sorption by coexisting cations. The inhibition of Na^+^ and Ca^2+^ to propranolol sorption suggests that the ion exchange plays an important part in propranolol sorption on MATP and HMATP. In addition, HMATP shows a higher sorption capacity than MATP in this condition, which suggests that coated HA takes part in the propranolol sorption on HMATP.

### 3.4. Effect of Sorbent Dosage

**Figure 9:**
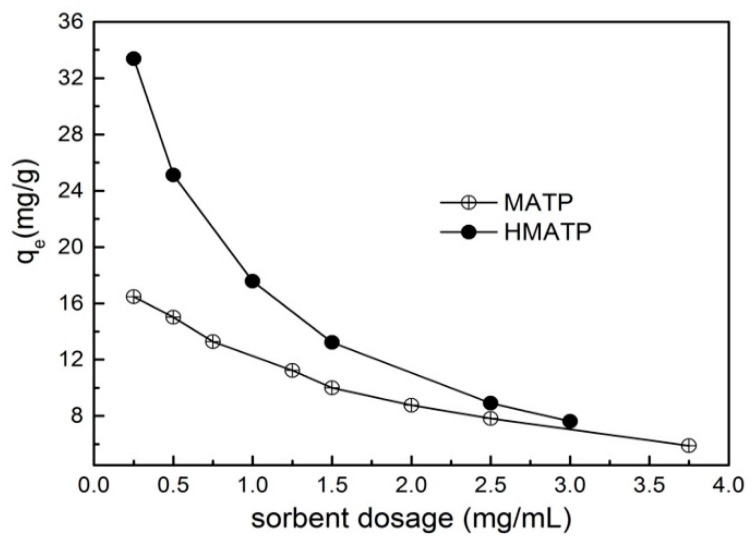
Effect of sorbent dosage on the sorption of propranolol on MATP and HMATP.

The relation curves between sorbent dosage and the sorption capacity are exhibited in Figure 9. It can be seen that the sorption capacities of propranolol on both MATP and HMATP decline with the increase in sorbent amount. With the increase of sorbent dosage, the sorption capacity of propranolol becomes less and less on unit sorbent dosage. In addition, the sorption capacity and the decrease rate of sorption capacity of HMATP are higher than those of MATP, which indicates that HA takes part in the propranolol sorption process.

### 3.5. Effect of HA

**Figure 10:**
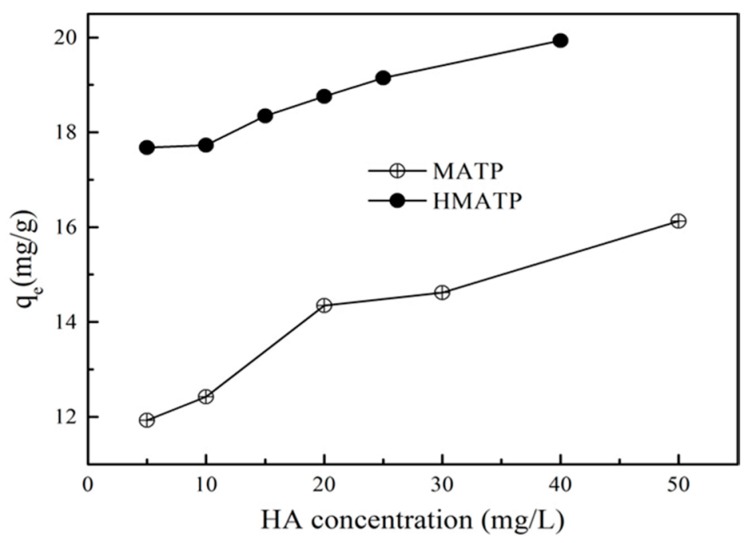
Effect of humic acid on the sorption of propranolol on MATP and HMATP.

Ubiquitous HA in environmental aquifers dramatically influences propranolol uptake on ATP [27], because low concentration of HA can be sorbed onto ATP [43]. The binding of HA directly on the sorption sites of ATP may inhibit the propranolol sorption because of the site blockage and competition if HA and propranolol occupy the same sorption sites [27]. To make clear the role of HA, the effect of different HA on propranolol sorption to MATP and HMATP were conducted. Figure 10 presents the results which show that propranolol sorption on MATP is enhanced with an increasing of HA concentration at about pH = 6. HMATP has higher sorption capacity than MATP at all tested conditions, which suggested that HA coated with MATP had same promotion to propranolol sorption. The more that HA binds with MATP, the higher the sorption capacity of propranolol on the mentioned magnetic nanocomposites, which may be explained by the introduction of many oxygen-containing functional groups in HA. Hydrogen bonding is formed in two ways: (1) N atoms of secondary amine of propranolol and H atoms of hydroxyl groups of HA. (2) H atoms of secondary amine of propranolol and O atoms of hydroxyl groups of HA. In addition, π–π electron donor accepter interaction (EDA) between the naphthalene rings structure of propranolol and the aromatic part of HA could also contribute to the sorption promotion. It can also be seen that the promotion slows down as HA concentration increased from 20 to 50 mg/L. HA molecules can occupy the sorption sites that also interact with propranolol, and space steric hinders the further contact of propranolol with the sorbents. Thus, sorption enhancement is, to some extent, offset by the inhibition. Nevertheless, HMATP still keeps higher sorption ability than MATP, which clearly demonstrates that surface-bound HA will increase propranolol sorption on MATP.

### 3.6. Sorption isotherms

The sorption isotherm describes the sorbent-sorbate relationship at equilibrium, critical in determining optimal parameters for the application of the sorbent [43]. The Langmuir (Equation (2)), Freundlich (Equation (3)), and Sips (Equation (4)) sorption equations were fitted with experimental equilibrium data. The Langmuir sorption model supposes that there are a finite number of identical sorption sites on the surface of sorbent and the surface is homogeneous. The Freundlich model is an empirical model which assumes that the surface of the sorbent occurs multilayer sorption. The Sips model is a further improvement of the Langmuir model and the Freundlich model.

(2) $$q_{e}=\frac{q_{m}K_{L}C_{e}}{1+K_{L}C_{e}}$$

(3) $$q_{e}=K_{F}C_{e}{}^{\frac{1}{n_{1}}}$$

(4) $$q_{e}=\frac{q_{m}{\left(bC_{e}\right)}^{\frac{1}{n_{2}}}}{1+{\left(bC_{e}\right)}^{\frac{1}{n_{2}}}}$$

*q_e_* and *q_m_* are the sorption capacity and maximum sorption capacity of propranolol, respectively; *C_e_* is propranolol sorption after sorption; and *K_L_* is the Langmuir coefficient, and *K_F_* is the Freundlich isotherm constant. 1/*n_1_* (dimensionless) is relative with the heterogeneity of the sorbent. 1/*n_2_* represents the heterogeneity of the sorbent. The value is closer to 1, the more uniform the surface of the sorbent. *b* is the median association constant.

**Figure 11:**
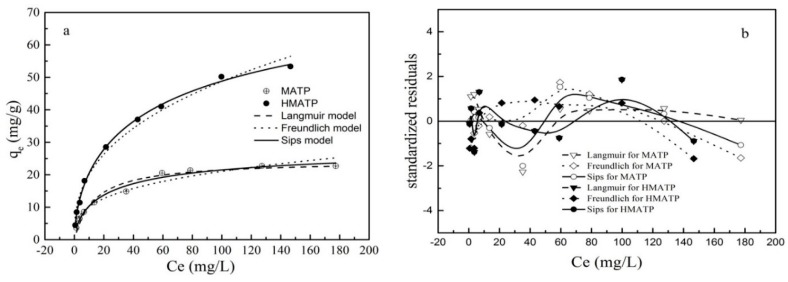
(**a**) Sorption isotherm of propranolol on MATP and HMATP. (**b**) Residual plot for data for Langmuir, Freundlich, and Sips models.

The experimental and fitted data of propranolol sorption onto MATP and HMATP are given in Figure 11a. According to reference [44], the standardized residuals of calculated data and experimental data are plotted in Figure 11b. As shown in the figure, the sorption capacity of propranolol increases when propranolol equilibrium concentration increases. The related calculated parameters are listed in Table 1. For MATP, the R^2^ of Sips model is higher than that of Langmuir and Freundlich models. The standardized residuals for the three models have similar distribution. It can be concluded that the sorption of propranolol on MATP is accorded with Sip model rather than Langmuir and Freundlich models, which indicates that the heterogeneous surface of MATP occurs during both the chemical and physical sorption processes. Compared with MATP, R^2^ is the same height for the three models but the residue plot of Sips model is closer to 0 than the other two models. The result shows that the Sips model explains the sorption process better than the other models, and indicates that multilayer sorption is formed on the heterogeneous surface sites of HMATP. Moreover, the n value, a Sips parameter, represents the degree of uniformity on the surface of the sorbent. Based on Table 1, the n value of HMATP is higher than that of MATP, indicating that the introduction of HA reduces the heterogeneity of the MATP surface.

### 3.7. Sorption Kinetics 

**Figure 12:**
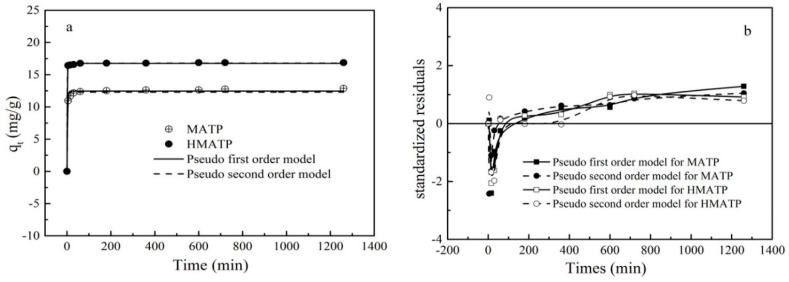
(**a**) Sorption kinetics of propranolol on MATP and HMATP. (**b**) Residual plot for data of fit values of pseudo first order model and pseudo second order model.

The relative curves between the sorption capacity of HMATP and MATP and contact time are shown in Figure 12. The rate of the sorption is very fast during the first 30 min of contact for HMATP and MATP, which may be attributed to a great deal of sorption sites that are available for propranolol. However, the sorption rate is slowed down after 1 h because of the sites saturation. To further investigate the sorption of propranolol on HMATP and MATP, the experimental kinetics data of propranolol sorption were simulated by the following pseudo-first-order rate models and pseudo-second-order rate models, respectively:

(5) $$q_{t}=q_{e}\left(1-e^{-kt}\right)$$

(6) $$q_{t}=\frac{kq_{e}{}^{2}t}{1+kq_{e}t}$$

where *q_e_* and *q_t_* correspond to the sorption capacity of propranolol at equilibrium and at time *t* (min), respectively. Both *K_1_* and *K_2_* are sorption rate constants.

The corresponding fitting results and the kinetics parameters are shown and listed in Figure 12 and Table 2, respectively. As shown in Figure 12a, the fitted curves of the pseudo-first-order kinetics and the pseudo-second-order kinetics are close to the experimental data. However, the R^2^ of the pseudo-first-order kinetics for MATP in Table 2 is slightly higher than that of the pseudo-second-order kinetics, which indicates that the pseudo-first-order kinetics is more in line with the experimental data than the pseudo-second-order kinetics. R^2^ of two models for HMATP is the same, which may be ascribed to the loading of HA. A residual plot for each set of data was drawn to further analyze the goodness of fit (Figure 12b). For MATP, the difference between the standardized residual distributions of the two fits is small. In contrast, for HMATP, the residual distribution of pseudo-second-order kinetics is closer to zero. Therefore, it is reasonable to infer that MATP is more in line with the pseudo-first-order kinetic equation and HMATP is fitted with the pseudo-second-order kinetic equation.

### 3.8. Material Stability 

**Figure 13:**
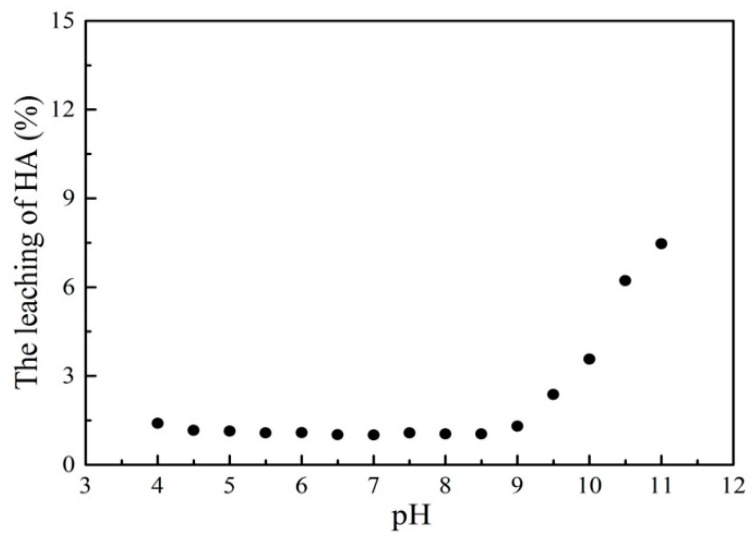
The leaching of HA after sorption at different pH levels.

To evaluate the stability of the HMATP nanoparticles in aqueous solution during the sorption reaction, the leaching of HA after sorption at different pH levels was determined (Figure 13). In the relatively large range of pH 4–9, the leaching of HA was kept at about 1.5% which is almost negligible. When the pH was greater than 9, the concentration of HA in the solution increased, which may be due to the dissolution of HA under the alkaline conditions. At the same time, the Fe leaching of HMATP was also measured. The results show that the concentrations of Fe leached are less than 0.017 mg/L under the condition of pH from 4 to 9, which are below the detection limit. The results indicate that HMATP nanoparticles display good stability when used to uptake propranolol from natural water.

### 3.9. Comparison of HMATP with Other Sorbents

To further evaluate the performance of the sorbent, the sorption capacity of HMATP (53.5 mg/g) was compared to other sorbents. In our previous work, acidified attapulgite, chitosan modified attapulgite, and coupling agent modified attapulgite were prepared as sorbents to remove propranolol from water, the sorption capacities of them were 48.05 mg/g, 26.38 mg/g, and 24.56 mg/g, respectively [27]. Compared with the three sorbents, HMATP can be easily separated from water. A similar material, magnetic graphene oxide (GO/Fe_3_O_4_), was prepared in a previous study [30]. The sorption capacity was about 55 mg/g which showed the same sorption ability as HMATP. However, difficult preparation and high cost make graphene oxide unsuitable for applications. María del Mar Orta et al. used montmorillonite as a sorbent to remove propranolol, which was found to have a sorption capacity of about 25.9 mg/g [42]. It was obvious that the sorption capacity of propranolol of HMATP was higher than the above sorbents. In summary, HMATP is a promising sorbent, which can be used to remove propranolol from water. 

## 4. Conclusions

In the present work, a novel sorbent, HMATP, was prepared to remove propranolol from water. Coating with HA introduced a number of oxygen-containing functional groups on MATP. HMATP maintained a high sorption capacity within a broad pH range. Ionic strength showed slight inhibition to propranolol sorption. HA in solution enhanced the sorption ability of HMATP to propranolol. HMATP had a better sorption ability than MATP in all the tested conditions, suggesting the promoting sorption of surface-coated HA. The experimental results indicated that electrostatic interaction and cation exchange are the main mechanisms for HMATP, because the main species of propranolol in solution was in cation form. Besides, hydrogen bonding and π–π EDA may play indispensable roles in the whole sorption process. Generally, it is feasible to use HMATP as a sorbent to remove propranolol from water, which provides a new insight to treat and remove beta-blockers from the environment.

## Figures and Tables

**Table 1:** Sorption isotherms parameters for propranolol sorption onto HMATP.

Materials	Langmuir Equation			Freundlich Equation			Sips Equation			
	*q_m_* (mg/g)	*K_L_* (L/mg)	*R* ^2^	*K_F_* (L/g)	*n*_1_	*R* ^2^	*q_m_* (mg/g)	*n*_2_	*b*	*R* ^2^
MATP	24.33	0.075	0.964	4.94	3.18	0.945	29.83	1.46	0.04	0.979
HMATP	92.42	0.072	0.997	8.33	2.61	0.990	92.42	1.68	0.01	0.997

**Table 2:** Kinetic parameters for propranolol sorption onto HMATP.

Materials	*q_exp_* (mg/g)	Pseudo-First-Order Equation			Pseudo-Second-Order Equation		
		*K*_1_ (1/min)	*q_cal_* (mg/g)	*R* ^2^	*K*_2_ (g/(mg·min))	*q_cal_* (mg/g)	*R* ^2^
MATP	12.87	0.419	12.463	0.992	0.094	12.291	0.976
HMATP	16.87	0.797	16.752	0.999	0.459	16.796	0.999
